# Immune-related histologic phenotype in pretreatment tumour biopsy predicts the efficacy of neoadjuvant anti-PD-1 treatment in squamous lung cancer

**DOI:** 10.1186/s12916-022-02609-5

**Published:** 2022-10-24

**Authors:** Pei Yuan, Changyuan Guo, Lin Li, Yun Ling, Lei Guo, Jianming Ying

**Affiliations:** Department of Pathology, National Cancer Center/National Clinical Research Center for Cancer/Cancer Hospital, Chinese Academy of Medical Sciences and Peking Union Medical College, No. 17, Panjiayuan Nanli, Chaoyang District, Beijing, 100021 China

**Keywords:** Neoadjuvant, PD-1, Immune-related histologic phenotype, Biopsy, Pathologic response

## Abstract

**Background:**

Although neoadjuvant anti-PD-1 immunotherapies have shown good efficacy in non-small cell lung cancer (NSCLC) patients, there is still a lack of effective predictive markers. We aimed to develop a pretreatment histologic scoring system to predict the efficacy of neoadjuvant immunotherapy.

**Methods:**

One hundred forty NSCLC cases were evaluated in this study. Initially, surgical specimens from 31 squamous cell lung cancer patients treated with neoadjuvant anti-PD-1 therapy and their eligible paired pretreatment biopsies were used for pathologic evaluation and developing the pretreatment scoring system, immune-related histologic phenotype assessment criteria (irHPC). Three trained pathologists independently scored the haematoxylin-eosin (HE) slides of the pretreatment tumour biopsies according to irHPC. The follow-up was from 07 March 2018 to 31 December 2021, mainly focusing on disease-free survival (DFS) and overall survival (OS). Second, 109 biopsies of lung squamous cell carcinoma were evaluated to explore the relationship between eosinophils and PD-L1 expression.

**Results:**

Superior 2-year DFS rates and 2-year OS rates were observed in patients who achieved major pathologic response (MPR) (MPR vs. non-MPR: 92.9% vs. 78.6%; 100.0% vs. 93.3%). Whether necrosis was included in the calculation of the per cent of residual viable tumour (%RVT) or not had almost no effect on the consistency of pathologic assessment and the histological response grouping. The interpathologist variability in assessing %RVT with immune-activated phenotype was not statistically significant (*P* = 0.480). Four immune-related features of pretreatment biopsies were included for calculating the predictive score. The trained pathologist accurately predicted most cases according to irHPC. For interobserver reproducibility using “2 points” as the cutoff, the overall per cent agreement was 77.8%. The reliability between pathologists for a binary tumour evaluation showed “moderate” agreement (*κ* = 0.54). Patients with scores ≥ 2 points tended to have better 2-year DFS rates and 2-year OS rates than those with scores < 2 points (85.7% vs. 71.4%; 100.0% vs. 87.5%).

**Conclusions:**

The irHPC scoring system reflecting the preexisting immune response could be used to predict pathologic response to neoadjuvant immunotherapy, possibly further predicting the long-term prognosis, but larger trials are needed for verification.

**Supplementary Information:**

The online version contains supplementary material available at 10.1186/s12916-022-02609-5.

## Background

The development and progression of tumours are always accompanied by interactions with the tumour microenvironment [1]. The distinct mechanism of immunotherapy, particularly the PD-1/PD-L1 immune checkpoint inhibitors, is to reactivate and enhance the existing adaptive immune response to indirectly kill the tumour [2]. Studies have shown that a variety of immune cells are involved in the immune response against tumours, including T cells, B cells, macrophages, and plasma cells [3]. However, most studies have explored them by means of gene expression profiling in bulk tumours [4]. Bulk tumour RNA based immune signatures simply reflect the average number of signals in a group of cells or only the information of the dominant cell subgroups but ignore the unique characteristics of individual cells. To a great extent, tumour heterogeneity is a hurdle to reproducibility and consistent results both within and across studies.

To overcome the hurdles of bulk tumour transcriptomics, ongoing single-cell RNA sequencing is becoming an alternative strategy [5, 6]; it characterizes the composition of the tumour immune microenvironment and reveals that the tumour immune microenvironment, including tertiary lymphoid structures (TLSs) and other subsets of immune cells, is critical to the immune response and can predict the effect of tumour immunotherapy. However, it is still challenging in clinical application [7].

Immunohistochemistry­ based methods have been used to assess the immunological status of tumours and enrich prognostic information. One example is Immunoscore, a digital pathology-based assay derived from the quantification of CD3^+^ lymphocytes (total tumour-infiltrating T cell counts) and CD8^+^ lymphocytes (cytotoxic tumour-infiltrating T cell counts) at the invasive margin and at the core of the tumour [8]. However, such methods, including multiplexed fluorescent immunohistochemistry techniques, are limited by problems with the standardization of testing and interpretation [9].

With the development of neoadjuvant immunotherapy trials, the neoadjuvant platform affords an unparalleled opportunity for reverse translation: surgical specimens obtained after neoadjuvant immunotherapy provide a rich source of materials for the morphological evaluation of the body’s immune response to tumour immunotherapy that are expected to further illuminate the mechanisms of action for anti-PD-1/PD-L1 drugs [10, 11]. Based on the preexisting immune response that exists in the process of tumour development and the morphological changes we mastered on the pathologic assessment after neoadjuvant immunotherapy, we hypothesize that there are morphological clues in tumour biopsy before neoadjuvant therapy that could indicate whether the necessary immune response exists and predict the efficacy of neoadjuvant immunotherapy.

In this study, we developed a novel pretreatment scoring system that reflects the preexisting immune response to explore the predictive effect regarding the pathologic response after neoadjuvant immunotherapy.

## Results

### Pathologic response and follow-up

Among 31 enrolled neoadjuvant patients, 15 (48.4%) patients achieved MPR. By data cutoff (31 December 2021), the median follow-up for DFS and OS for all the enrolled patients was 36.9 (18.6–45.2) months and 38.3 (19.9–46.5) months, respectively. Seven (22.6%) patients experienced relapse, four patients died, and two patients whose deaths were not related to the treatment were excluded from the survival analysis. The 1-year and 2-year DFS rates were 96.6% and 85.8%, respectively. The 2-year OS rate for all enrolled patients was 96.6%. The 3-year survival data were the same as the former. Superior 2-year DFS rates and 2-year OS rates were observed in patients who achieved major pathologic response (MPR) (MPR vs. non-MPR: 92.9% vs. 78.6%; 100.0% vs. 93.3%) (Additional file 1: Table S1).

### Percent of residual viable tumour (%RVT) with necrosis versus without necrosis

**Fig. 1:**
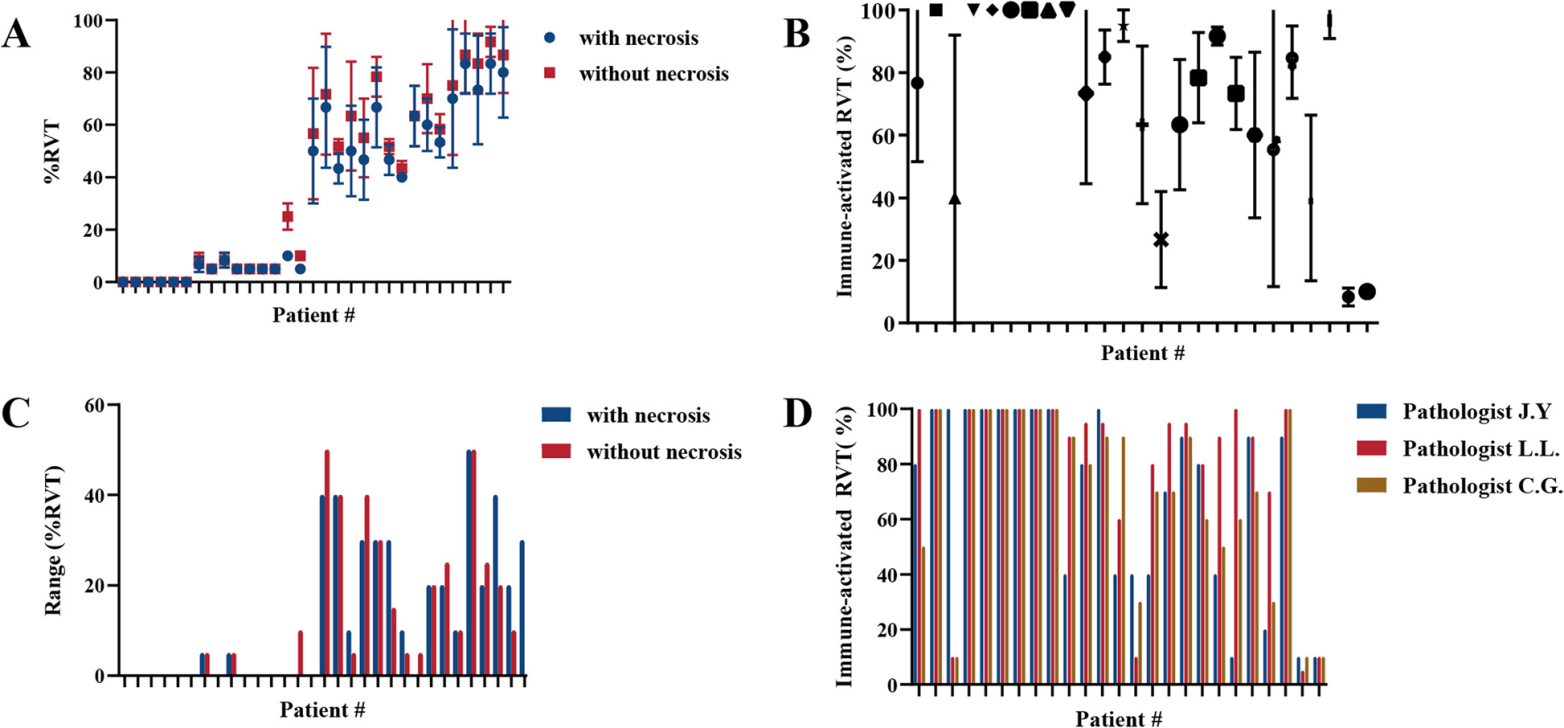
Histopathologic assessment of response to treatment in resected samples: the influence of the calculation of %RVT with and without necrosis and emphasizing the assessment of the histologic phenotype of RVT cells. Whether %RVT was calculated with or without necrosis had almost no effect on the consistency of pathologic assessment and the pathologic response group, except for patient 14, whose group changed from MPR to partial pathologic response (pPR) (**A**) with a mean value changing from 10 to 25% (**C**). The mean ± SD of %RVT with immune-activated phenotype for each case is shown in **B**. The interpathologist variability of assessing %RVT with immune-activated phenotype was not statistically significant (*P* = 0.480) (**D**)

Necrosis was not common in resected specimens with complete pathologic response (cPR) after neoadjuvant anti-PD-1 treatment in our cohort (1/6). In 31 surgical specimens, the calculation of %RVT with or without necrosis resulted in changing the pathologic response group in only one case (patient 14, the mean value changed from 10 to 25%, Fig. 1A), although %RVT-without necrosis was higher in 17 of 31 cases compared with that with necrosis (*P* < 0.001, range: 5–20%). Whether necrosis was included in the calculation of %RVT or not had no effect on the consistency of pathologic assessment (Fig. 1C, *P*=0.811).

### Intratumor heterogeneity of pathologic response to neoadjuvant anti-PD-1 treatment

**Fig. 2:**
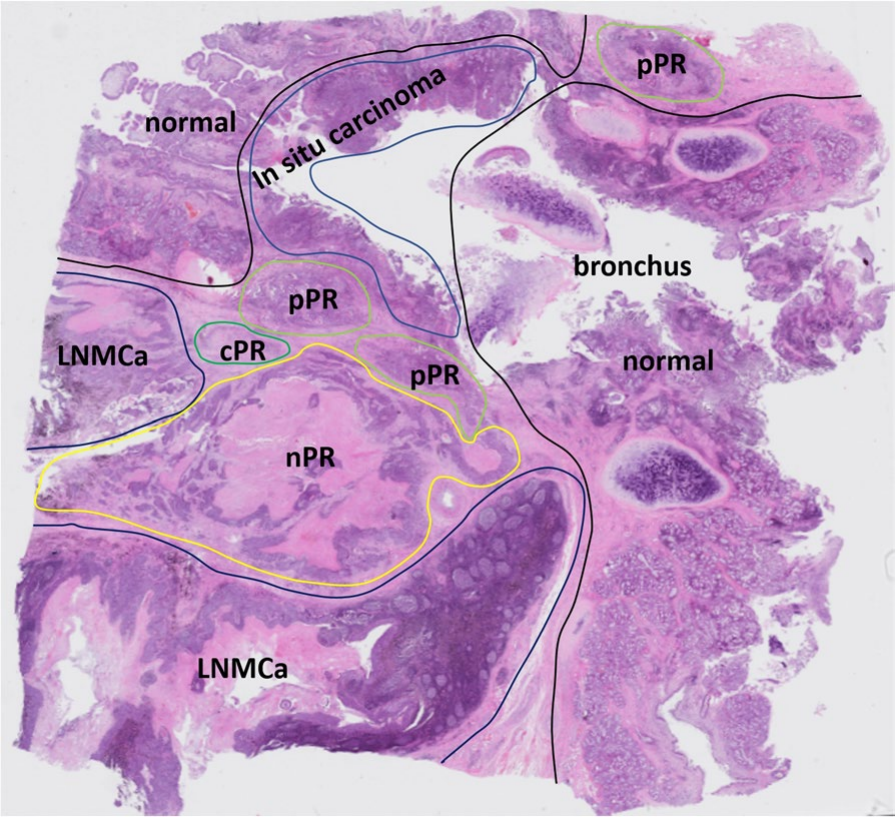
Intratumor heterogeneity of pathologic response to neoadjuvant anti-PD-1 treatment: different responses of tumour cell subclones in the same tumour bed. cPR, complete pathologic response; pPR, partial pathologic response; nPR, no pathologic response; LNMCa, lymph node metastatic carcinoma

**Fig. 3:**
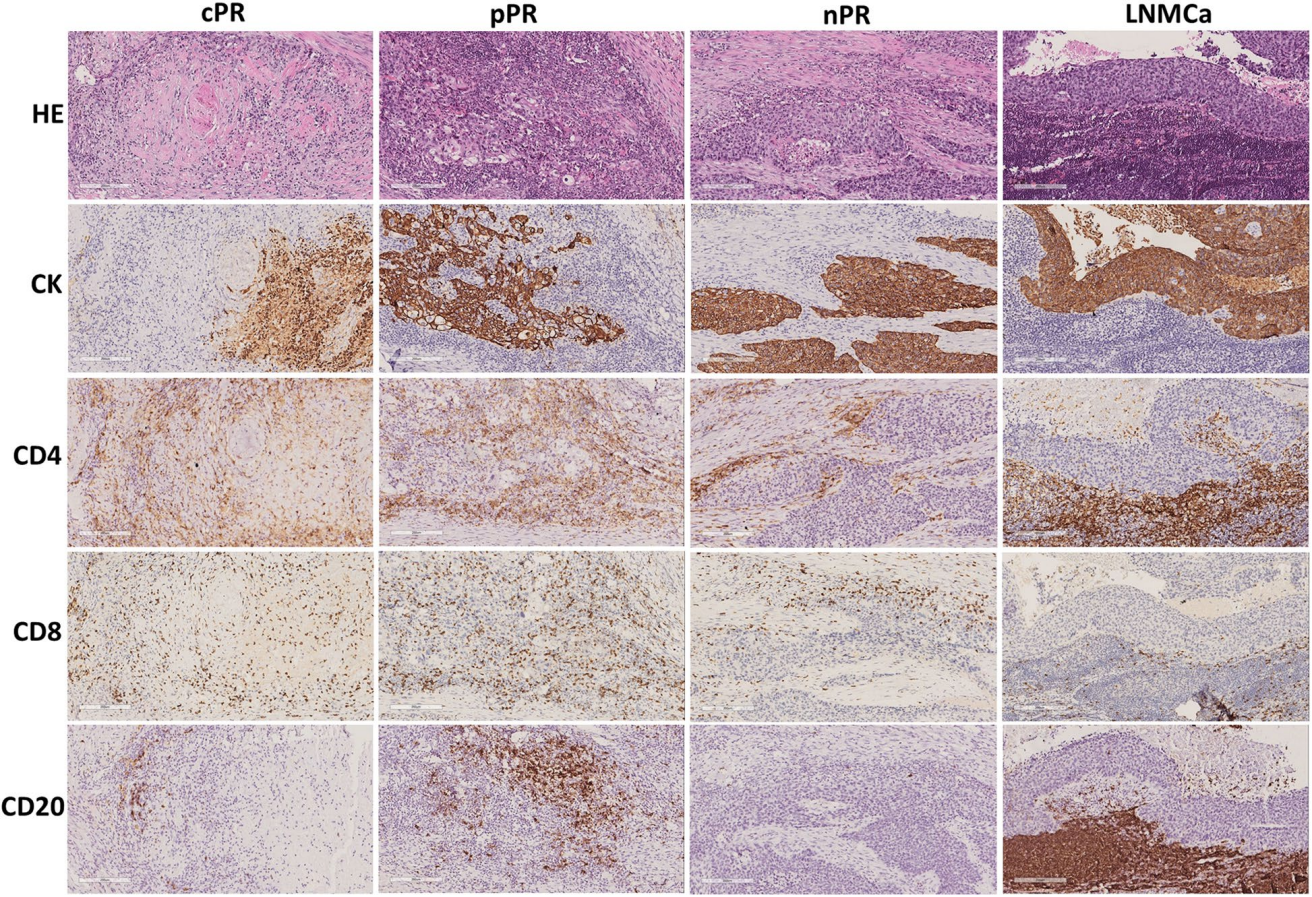
Histological morphology and immune cell distribution in different response regions. CK staining showed the tumour nests, and CD4, CD8, and CD20 showed the infiltrated immune cells: CD4^+^ T cells, CD8^+^ T cells, and CD20^+^ B cells. cPR, complete pathologic response; pPR, partial pathologic response; nPR, no pathologic response; LNMCa, lymph node metastatic carcinoma

**Fig. 4:**
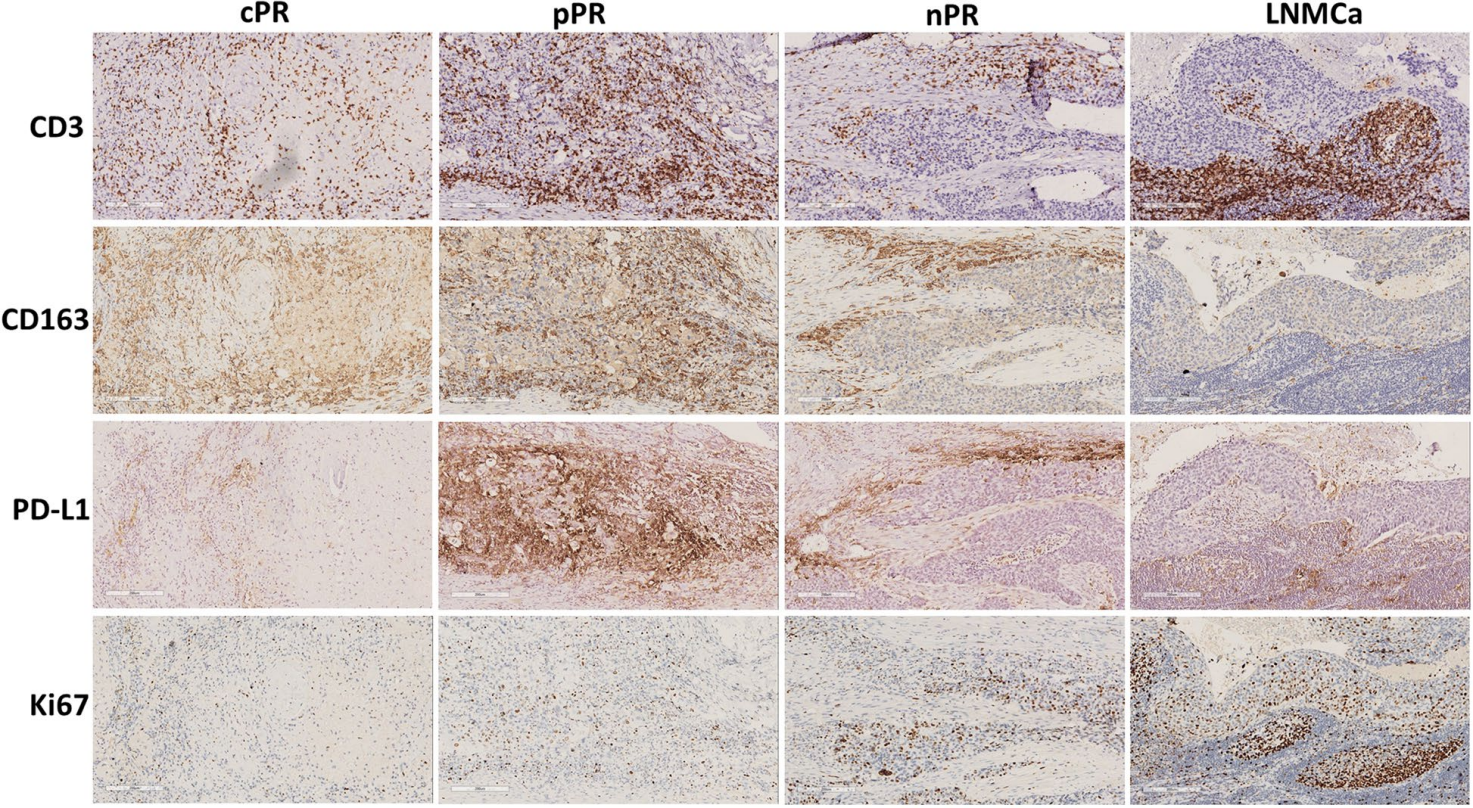
Immune infiltration and the expression of PD-L1 in different response regions. CD3 and CD163 showed infiltrated CD3^+^ T cells and histocytes. cPR, complete pathologic response; pPR, partial pathologic response; nPR, no pathologic response; LNMCa, lymph node metastatic carcinoma; PD-L1, programmed cell death ligand-1

Due to intratumor heterogeneity of either genetics or the tumour microenvironment, the response of tumour cell subclones in the same tumour bed to PD-1 inhibitors is different, and the time required for tumour cell clearance varies (Fig. 2). We also identified three histologic phenotypes of RVT cells after anti-PD-1 immunotherapy: immune-activated phenotype, immune-excluded phenotype, and immune-desert phenotype. In addition to CD8^+^ T cells, we also characterized CD4^+^ T cells, CD20^+^ B cells, and CD163^+^ histocytes infiltrating the tumour-related stroma (Figs. 3 and 4).

### Interpathologist reproducibility in assessing immune-activated histologic phenotype

The immune-activated histologic phenotype is characterized by the presence of immune cells both in the parenchyma of the tumour nest and the surrounding stroma and is accompanied by different levels of irregular tumour-stroma interface. Twenty-five cases with incomplete remission all had different degrees of RVT with immune-activated histologic phenotype, in which eight of nine MPR specimens had 100% of immune-activated phenotype RVT, and the remaining specimen had 80%. The interpathologist variability in assessing the per cent of RVT with immune-activated phenotype was not statistically significant (*P* = 0.480), especially for the MPR cases, in which seven of nine cases were identical under the assessments of all three pathologists (Fig. 1B, D).

### immune-related pathologic response criteria (irHPC): a pretreatment immune-related histologic phenotype assessment system

Compared with the pathologic response, four immune-related features of pretreatment biopsies were included for calculating the predictive score, including three positive features [tumour infiltrating lymphocytes (TILs), eosinophils, and dense plasma cells] and one negative feature (neutrophils). The score was defined according to the degree of infiltration of TILs, eosinophils, and neutrophils and whether the dense plasma cells existed or not: 0, 1+, 2+, and 3+ of TILs or eosinophils were equal to 0, 1, 2, and 3 points, respectively, but a negative score was assigned for the neutrophils; if dense plasma cells existed in the tumour-related stoma, 1 point was scored, otherwise 0. Then, the four scores were added to obtain the final predictive score.

### irHPC in pretreatment tumour biopsy predicts the efficacy of neoadjuvant anti-PD-1 treatment

According to the reference scores, the majority of cPR/MPR cases (87.5%, 7/8) scored no less than 2 points (range: 2 to 5 points), except for one case with 0 points (patient 13), whose pretreatment tumour size was 2.2 cm. All pPR/no pathologic response (nPR) cases (100%, 7/7) scored less than 2 points (range: − 2 to 1 point). Survival analysis showed that patients with scores ≥ 2 points tended to have better 2-year DFS rates and 2-year OS rates than those with scores < 2 points (85.7% vs. 71.4%; 100.0% vs. 87.5%) (Additional file 1: Table S1). We speculated that the possible predictive cutoff might be 2 points. Out of the 15 patients, the trained pathologist (C.G.) accurately predicted six out of eight patients in the cPR/MPR group and five out of seven patients in the pPR/nPR group. The other two pathologists accurately predicted four out of five patients and six out of ten patients in the predicted cPR/MPR group and pPR/nPR group, respectively (Additional file 2: Table S2). There were 35 concordant pairs, resulting in an overall per cent agreement (OPA) of 77.8%. The reliability between pathologists for a binary tumour evaluation showed “moderate” agreement at the “2 points” cutoff (*κ* = 0.54) (Additional file 3: Table S3).

There were three recurrence cases among these 15 cases, including one MPR case and two pPR cases. These three cases were scored by four pathologists, and there were at least two pathologists predicting less than 2 points for each case.

### The derived neutrophil-to-lymphocyte ratio (dNLR) supports the negative predictive role of neutrophils

There were 10 cases with a dNLR greater than 3, including seven pPR/nPR cases and three cPR/MPR cases. Among 15 cPR/MPR cases, 12 cases had dNLR values that were no greater than 3 (Additional file 4: Table S4). For the 15 cases with paired pretreatment biopsies, there were five cases with a dNLR greater than 3, four of which were pPR/nPR cases and three of which were found to have extensive neutrophils in the parenchyma of the tumour in the pretreatment biopsies. Survival analysis showed that inferior 2-year DFS rates and 2-year OS rates were found in the patients with a dNLR > 3 (dNLR > 3 vs. dNLR ≤ 3: 80.0% vs. 89.2%; 90.0% vs. 100.0%).

### The relationship between eosinophils and PD-L1 expression

Regarding PD-L1 expression, on the basis of the described scoring procedure, 108 cases were available for evaluation, with 1 case excluded because of insufficient tumour cells on the immunohistochemical section. There were 59 cases with tumour proportion score (TPS) ≥ 1%, 16 of which had TPS ≥ 50% (Additional file 5: Table S5).

Regarding eosinophils, there were 45 cases with eosinophils appearing in both the parenchyma of the tumour and the stroma, and the level of PD-L1 expression was higher than in the other 63 cases (12 cases and 4 cases with TPS ≥ 50%, respectively, *P* = 0.003); however, there was no significant difference for the cutoff point of 1% (27 cases and 32 cases, respectively, *P* = 0.343) (Additional file 5: Table S5).

## Discussion

Neoadjuvant anti-PD-1 immunotherapies have shown good efficacy in non-small cell lung cancer (NSCLC) patients, but there is still a lack of effective predictive markers. Neoadjuvant checkpoint blockade for cancer immunotherapy has highlighted particular considerations for pathologic response assessment in the era of PD-1 pathway blockade [12, 13]. In this study, we further explored some questions about the pathologic evaluation of surgical specimens after neoadjuvant immunotherapy and for the first time proposed a pretreatment scoring system based on a morphological evaluation to predict the efficacy of neoadjuvant immunotherapy.

Although a standardized approach similar to neoadjuvant chemotherapy was recommended by the International Association for the Study of Lung Cancer to improve consistency in the pathologic assessment of neoadjuvant treatment response [12], we introduced some new ideas in our previous publications [14, 15], one of which was regarding necrosis. We previously assessed 31 surgical specimens of squamous cell lung cancer patients treated with neoadjuvant anti-PD-1 therapy according to the irPRC [14, 16]. In our assessment, immunotherapy-related necrosis was not common after neoadjuvant immunotherapy [15]. In the current study, although the per cent RVT-without necrosis was higher than that with necrosis in more than half of the cases, there was almost no effect on the pathologic response grouping and the consistency of per cent assessment among pathologists. Our present prognostic data showed that the removal of necrosis was not associated with patients’ outcomes. Considering the infrequency of necrosis in neoadjuvant immunotherapy, the inclusion of necrosis may overestimate the efficacy of neoadjuvant anti-PD-1 treatment efficacy (as in case 14 in our study, RVT changed from 10 to 25% without necrosis being accounted for) and mislead postsurgical treatment decision-making for individual patients. Therefore, whether necrosis is defined as the main factor in the pathologic evaluation after neoadjuvant immunotherapy remains to be further discussed.

In addition, we also identified three histologic phenotypes of the RVT cells after the anti-PD-1 immunotherapy: immune-activated phenotype, immune-excluded phenotype, and immune-desert phenotype. In a previous publication, we emphasized the potential importance of assessing immune-related phenotypes of RVT, particularly the immune-activated histologic phenotype [14]. In this study, we explored the interpathologist reproducibility in assessing immune-activated histologic phenotype, and the results showed that there was good consistency of interpretation among pathologists; in addition, for non-MPR cases, inferior 2-year DFS rates and 2-year OS rates were observed in cases where the immune-excluded/desert phenotype RVT was present compared with those in which it was absent (76.9% vs. 100.0%; 92.9% vs. 100.0%), further emphasizing the feasibility and importance of the evaluation of the histologic phenotype of the RVT immune response.

PD-L1 and tumour mutation burden (TMB) detection are used to screen the potential beneficiaries of immune checkpoint inhibitors for advanced NSCLC in clinical practice; however, the predictive efficacy of PD-L1 and TMB as pretreatment markers remains controversial [10, 11, 17, 18]. Labelling with simple immunohistochemical markers revealed that in addition to CD8^+^ T cells, a variety of immune cells are involved in the tumour immune process, which can also easily be observed with HE sections. Therefore, after hypothesizing that there are morphological clues in tumour biopsy before neoadjuvant therapy that could reflect whether the immune response exists and predict the efficacy of immunotherapy, we developed the irHPC scoring system.

Compared with the pathologic response, four immune-related features of pretreatment biopsies were included for calculating the predictive score, including three positive features (TILs, eosinophils, and dense plasma cells) and one negative feature (neutrophils). The latter is consistent with recent research [19, 20], in which the presence of neutrophils in tumours has been associated with poor efficacy of immune checkpoint inhibitors and poor prognosis. In 31 enrolled cases, most cases with pretreatment dNLR greater than 3 did not reach MPR, and for the 15 cases with matching pretreatment biopsies, there were four pPR/nPR cases with dNLR greater than 3 and three of which were found to have extensive neutrophils in the parenchyma of the tumour, which also supported the negative predictive role of neutrophils. For the three positive features, CD8^+^ TILs and plasma cells have been previously indicated as positive predictive factors with a protective role in antitumour immunity [21, 22]. For eosinophils, the review showed that different phenotypes were associated with different environmental stimuli, which could be tumour-promoting or tumour-suppressive [23]; however, in our study, we found that the presence of eosinophils in the parenchyma of the tumour was associated with the high PD-L1 expression (TPS ≥ 50%), which indicated that eosinophils seemed to have a synergistic positive predictive effect with PD-L1, supporting its predictive value. Like eosinophils, neutrophils used in the evaluation system also have double phenotypes, so we cannot determine that a single indicator is a tumour-promoting or tumour-suppressive [23–25]. For example, we proposed that neutrophils infiltrated in the tumour play a negative regulatory role in the tumour immune response. We found that there were one cPR/MPR case and two pPR/nPR cases with neutrophil infiltration. The former had a large number of TILs and a low degree of eosinophils and plasma cell infiltration, while the latter two were accompanied by only a few TILs and/or eosinophils. Therefore, the comprehensive score of the four indicators may reflect a balance between tumour promotion and tumour suppression, thus predicting the potential efficacy of neoadjuvant immunity. However, the precise signals and mechanisms involved still need to be further studied.

Although we highlight the advantages of neoadjuvant immunotherapy, for nonresponders, the neoadjuvant period might delay the treatment and miss potential curative surgery. Therefore, in addition to screening potential beneficiaries of neoadjuvant immunotherapy, the challenge is how to early identify patients who are unlikely to respond.

Synthesizing the relationship among the predictive score, the pathologic response, and prognostic data, we speculate that the possible predictive cutoff regarding whether neoadjuvant immunotherapy will be effective may be 2 points. In this study, three pathologists trained in irHPC assessed the 15 pretreatment biopsies. Although there were different extents of variability in the four score-related pathologic features, the trained pathologist accurately predicted most cases. Our follow-up data further showed that superior 2-year DFS rates and 2-year OS rates were observed in patients who achieved MPR and patients with irHPC scores ≥ 2 points, which was not due to the UICC VIII stages (Additional file 6: Table S6). In this study, there were at least two pathologists predicting less than 2 points for each of the three recurrence cases, which means that we can use irHPC to accurately predict not only the potential beneficiaries of neoadjuvant immunotherapy, but also the nonresponders to some extent, and further predicting the long-term prognosis. The interpretative pathologists need to have a solid morphological foundation, and this requirement may make the pretreatment scoring system, irHPC, more reliable and predictive of the pathologic response. In addition, due to the improved efficacy of the neoadjuvant combination treatment, an increasing number of cases were treated with a combination of immunotherapy and chemotherapy rather than neoadjuvant immunotherapy alone, so we were unable to study enough patients receiving neoadjuvant immunotherapy alone to verify the scoring system now. However, we tried to use 45 cases treated with neoadjuvant combination treatment as an external set to verify the predictive performance: out of the 45 cases, nearly 70% were accurately predicted using the irHPC, among which 14 out of 18 patients in the predicted cPR/MPR group (predictive scores no less than 2) were actual cPR/MPR cases (data not shown). However, the irHPC scoring system and the predictive cutoff point still need to be verified in a larger study.

Of course, the current predictive system is still unable to fully predict the potential pathologic response. One MPR case with a radiographic tumour size of 2.2 cm (patient 13) was incorrectly predicted under the scoring system, which demonstrated that the pretreatment assessment should combine the histologic phenotype with the radiographic tumour size. The latter has been illuminated in a former study [26], in which baseline tumour burden was an independent prognostic factor for the overall survival. Due to the small size, the clinical and/or biological characteristics (sex, smoking status, baseline PD-L1 expression, and TMB) of cases with different efficacies were not significantly different (data not shown). In our current exploratory study, we aimed to propose the possibility of predicting the efficacy of neoadjuvant immunotherapy based on baseline immune status. To establish a reliable prediction model, a larger cohort is needed.

## Conclusions

The surgical specimens obtained after neoadjuvant immunotherapy afford an unparalleled opportunity for reverse translation, which allowed the development of the irHPC scoring system; the latter can predict pathologic response after neoadjuvant immunotherapy, but larger trials are needed for verification. In addition, for pathologic evaluation after neoadjuvant immunotherapy, whether necrosis is defined as the main factor in pathologic evaluation after neoadjuvant immunotherapy remains to be further discussed. Furthermore, going beyond %RVT, the histologic phenotype of RVT cells may be of great significance in pathologic response to neoadjuvant immunotherapy.

## Methods

### Study design and patients

**Fig. 5:**
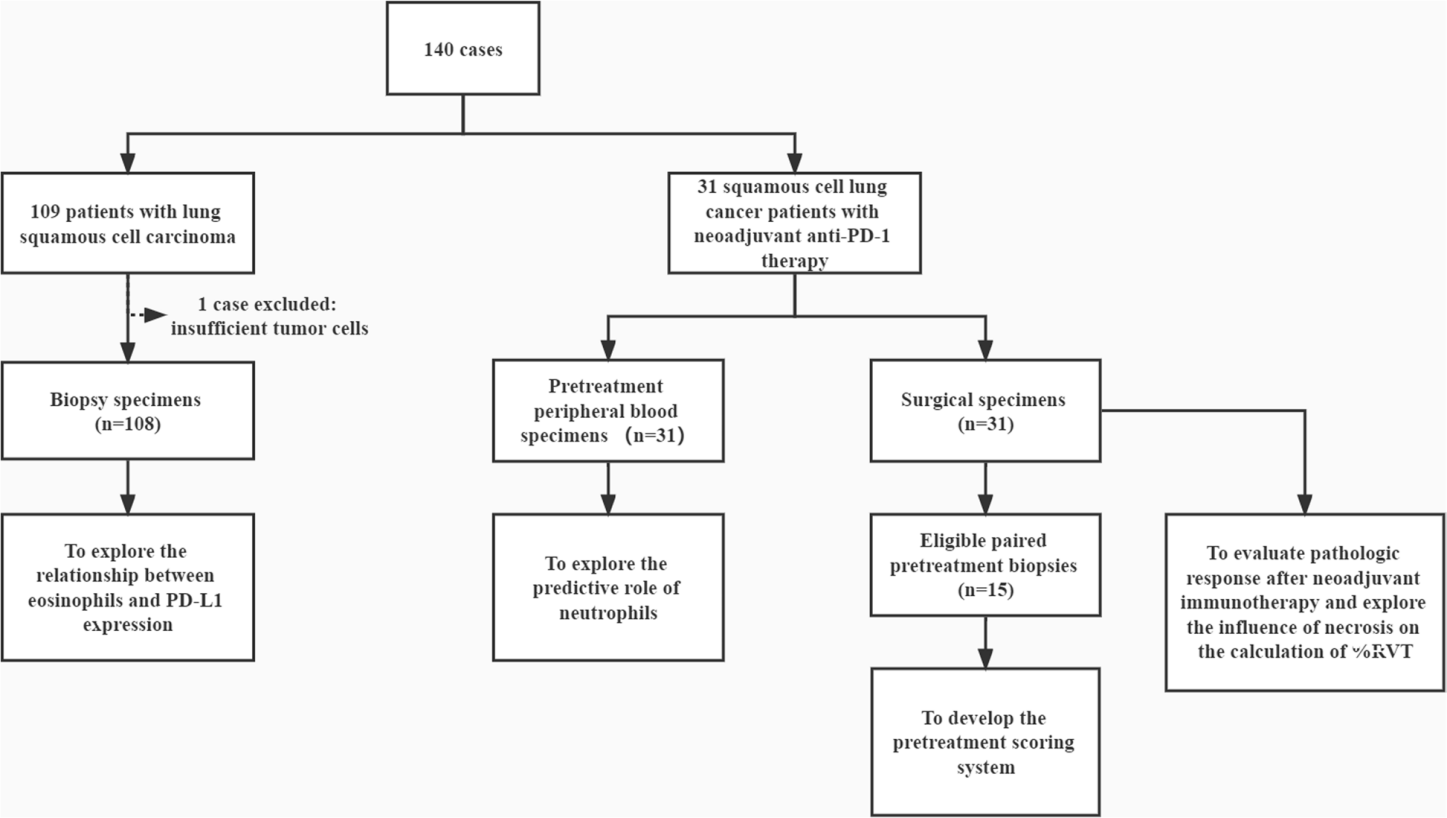
Flow diagram revealing the study design. %RVT, per cent of residual viable tumour

As the recruiting scheme shows (Fig. 5), surgical specimens from the 31 squamous cell lung cancer patients who were recruited in a phase 1b study of neoadjuvant anti-PD-1 therapy at the National Cancer Center/Cancer Hospital, Chinese Academy of Medical Sciences, Beijing (Registration Number: ChiCTR-OIC-17013726), and eligible paired pretreatment biopsies from 15 of them were included in this study. The 31 surgical specimens were used for pathologic evaluation after neoadjuvant immunotherapy and for exploring the influence of necrosis on the calculation of %RVT. The 15 pretreatment biopsies and paired surgical specimens were used to develop the pretreatment scoring system. The follow-up was from 07 March 2018 to 31 December 2021 and focused mainly on the survival time, including disease-free survival (DFS) and overall survival (OS).

To explore the relationship between eosinophils and PD-L1 expression, we collected biopsy specimens from another 109 patients with lung squamous cell carcinoma receiving care at Cancer Hospital, Chinese Academy of Medical Sciences, from January 2016 to May 2018.

### Derived neutrophil-to-lymphocyte ratio (dNLR)

The pretreatment-derived neutrophil-to-lymphocyte ratio (dNLR) has been considered a prognostic predictor for patients with NSCLC treated with immunotherapy [27]. Thirty-one squamous cell lung cancer patients had complete blood cell counts measured before treatment (Fig. 5). The dNLR was calculated as the derived neutrophils/(leukocytes minus neutrophils) ratio and evaluated on the basis of whether it was greater than 3 or not.

### Histopathologic assessment of response to treatment in resected samples

Haematoxylin-eosin (HE) slides containing tumour bed were all reviewed for semiquantitative evaluation of the percentage of viable tumour cells, necrosis, and regression bed according to the immune-related pathologic response criteria (irPRC) [16]. The per cent of residual viable tumour (%RVT) was calculated across all slides: %RVT = [total RVT area/total tumour bed area (total RVT area + necrosis + regression bed)] × 100%. The histological response was based on %RVT, and the following groups were defined: complete pathologic response (cPR, 0% RVT), major pathologic response (MPR, 1–10% RVT), and partial pathologic response (pPR, 11–90% RVT) or no pathologic response (nPR, > 90% RVT).

To explore the influence of necrosis on the consistency of pathologic assessment, we also calculated the per cent of RVT without necrosis (%RVT-without necrosis): %RVT-without necrosis = [total RVT area/(total RVT area + regression bed)] × 100%.

### Immune-related histologic phenotype assessment criteria (irHPC): a pretreatment scoring system

**Fig. 6:**
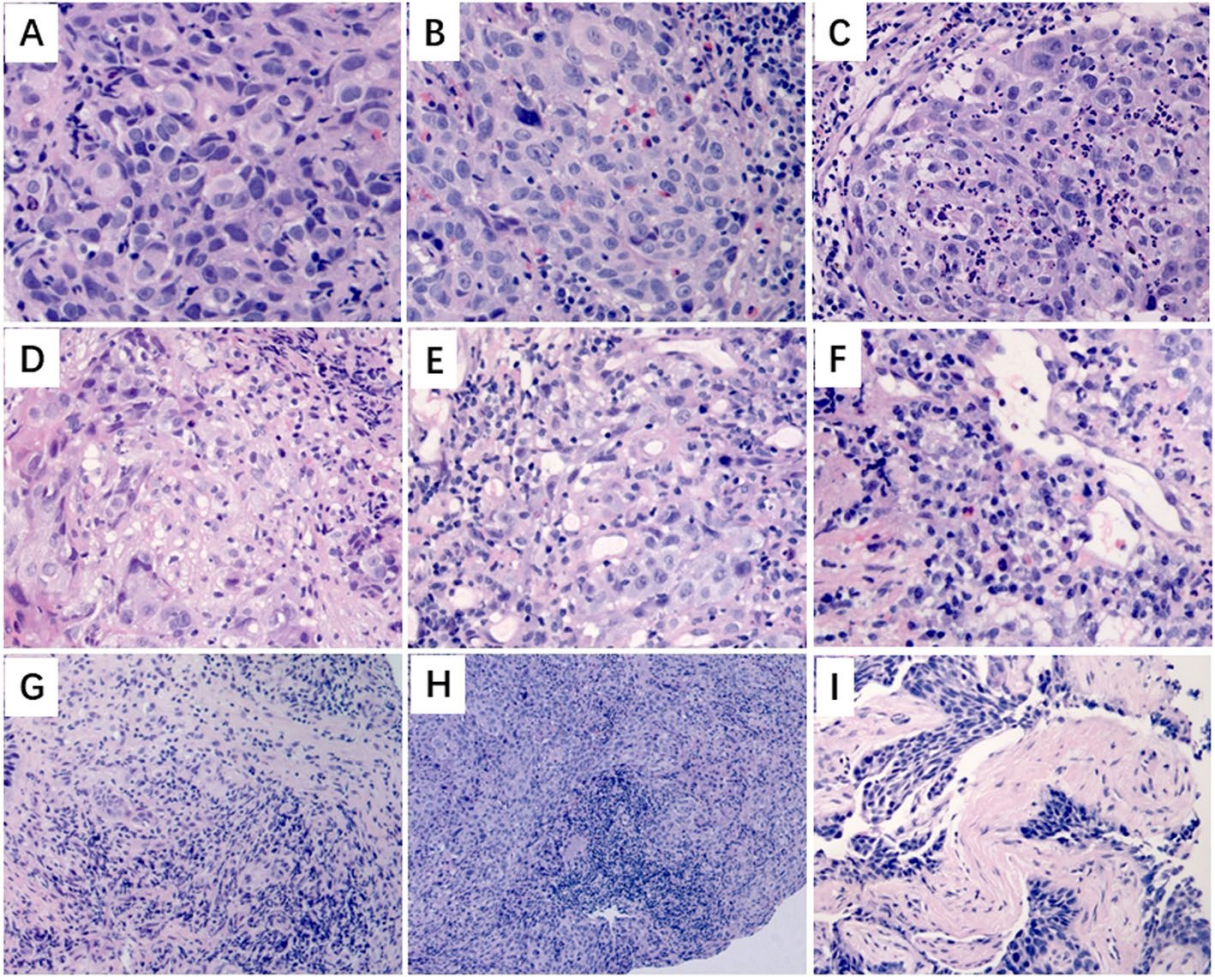
Immune-related histologic phenotype assessment criteria (irHPC). **A** Tumour-infiltrating lymphocytes (TILs). **B** Eosinophils. **C** Neutrophils. **D** Macrophages. **E** Irregular interface. **F** Dense plasma cells and neovascularization. **G** Lymphoid aggregate and proliferative (new) fibrosis. **H** TLSs. **I** Mature fibrosis

The irHPC scoring system was developed using the features associated with response to anti-PD-1 treatment, which were identified in the resected specimens after neoadjuvant anti-PD-1 treatment. In this scoring system, we assessed the immune status of the parenchyma of the tumour [tumour-infiltrating lymphocytes (TILs), eosinophils, neutrophils, and macrophages], the parenchyma-stroma interface (irregular interface), and the stroma of the tumour (dense plasma cells, lymphoid aggregate, proliferative fibrosis, neovascularization, TLSs, mature fibrosis, and necrosis) (Additional file 7: Supplemental methods, Fig. 6). The reference scores were defined by the pathologist (J.Y.) who was the key participator in the irHPC development.

### Histopathologic assessment of pretreatment tumour biopsies

Three pathologists (L.L., P.Y., and C.G.) not involved in the irHPC development were trained on irHPC using resection specimens and two other biopsies from patients not involved in the neoadjuvant immunotherapy trial. Each pathologist blinded to patient outcome and pathologic response then independently scored the HE slides from the 15 pretreatment tumour biopsies for preexisting immune-related histologic features according to irHPC.

### Immunohistochemistry

The formalin-fixed paraffin-embedded tissue blocks were serially cut into 4-μm sections, and the paraffin-embedded sections were deparaffinized, rehydrated, pretreated for antigen retrieval, and stained with ready-to-use primary antibodies as follows on Ventana BenchMark Ultra or DAKO Link48 Autostainer with respective detection kits: CD3 (Clone SP7, Kit-003, Maxim Biotechnologies, Fuzhou, China), CD4 (Clone UMAB64, ZM-0418, Zhongshan Golden Bridge Biotechnologies, Beijing, China), CD8 (Clone SP16, ZA-0508, Zhongshan Golden Bridge Biotechnologies, Beijing, China), CD20 (Clone L26, Kit-001, Maxim Biotechnologies, Fuzhou, China), CD163 (Clone 10D6, ZM-0428, Zhongshan Golden Bridge Biotechnologies, Beijing, China), CK (Clone AE1/AE3, Kit-009, Maxim Biotechnologies, Fuzhou, China), and PD-L1 (Clone 22C3 pharmDx, SK006, DAKO).

### Scoring of PD-L1 expression

Only the cases for which there were at least 100 viable tumour cells on each slide could be assessed. Staining of any intensity that was complete or partial on the tumour membrane (at a level ≥ 1%) was considered positive and was scored in terms of tumour proportion score (TPS). The results were analysed on the basis of two cutoff points, 1% and 50%.

### Statistical analysis

Statistical analysis was carried out using SPSS version 23.0 (IBM Corp) and Prism version 5.0 (GraphPad). The Wilcoxon signed ranks test was used to assess whether %RVT-without necrosis could reduce the differences among pathologists, and the Kruskal-Wallis test was used to analyse the consistency of immune-activated RVT% assessments among pathologists. The overall per cent agreement (OPA) and the 95% confidence interval (95% CI) were used to assess the interobserver reproducibility. The reliability between pathologists for binary tumour evaluation with the specific cutoff point was assessed by Fleiss’ kappa (*κ*), interpreted as poor to fair (≤ 0.40), moderate (0.41–0.60), substantial (0.61–0.80), and almost perfect (0.81–1.00 )[28]. The Kaplan-Meier method was used to estimate DFS and OS. All tests were two-sided, and *P* values of < 0.05 were considered statistically significant.

## Supplementary Information

**Additional file 1: Table S1.** DFS and OS for 29 neoadjuvant patients after surgery.

**Additional file 2: Table S2.** Different pathologists’ pretreatment prediction of pathologic response after neoadjuvant anti-PD-1 treatment according to irHPC.

**Additional file 3: Table S3.** Interobserver reproducibility of the irHPC scoring system to predict the pathologic response.

**Additional file 4: Table S4.** Derived neutrophil-to-lymphocyte ratio (dNLR) supports the negative predictive role of neutrophils.

**Additional file 5: Table S5.** The relationship between eosinophils and PD-L1 expression.

**Additional file 6: Table S6.** Comparison of UICC VIII stages between MPR and non-MPR cases.

**Additional file 7:** **Supplemental methods.** irHPC: a pretreatment scoring system.

## Data Availability

The data and materials that support the findings of this study are available from the corresponding author (Dr. Jianming Ying, email address: jmying@cicams.ac.cn.) upon reasonable request.
