# Supplementary material for: Immune-related histologic phenotype in pretreatment tumour biopsy predicts the efficacy of neoadjuvant anti-PD-1 treatment in squamous lung cancer
Source: BMC Med. 2022 Oct 24;20:403. doi: 10.1186/s12916-022-02609-5 (PMC9594940; doi:10.1186/s12916-022-02609-5)
Supplement: Supplementary file 1 — Additional file 1: Table S1. DFS and OS for 29 neoadjuvant patients after surgery. [file 12916_2022_2609_MOESM1_ESM.docx]

**Table S1 DFS and OS for 29 neoadjuvant patients after surgery**

| **Groups** | **N (event)** | **1-year DFS rate** | **2-year DFS rate** | **N (event)** | **2-year OS rate** |
| --- | --- | --- | --- | --- | --- |
| **irHPC score** |  |  |  |  |  |
| <2 | 8 (2) | 100.0% | 71.4% | 8 (2) | 87.5% |
| ≥2 | 7 (1) | 85.7% | 85.7% | 7 (0) | 100.0% |
| **Pathologic response** |  |  |  |  |  |
| non-MPR | 15 (4) | 100.0% | 78.6% | 15 (2) | 93.3% |
| MPR | 14 (1) | 92.9% | 92.9% | 14 (0) | 100.0% |
| **Non-MPR / Immune excluded/desert RVT (Primary tumour)** |  |  |  |  |  |
| Presence | 14 (4) | 100.0% | 76.9% | 14 (2) | 92.9% |
| Absence | 1 (0) | 100.0% | 100.0% | 1 (0) | 100.0% |
| **dNLR** |  |  |  |  |  |
| >3 | 10 (2) | 100.0% | 80.0% | 10 (2) | 90.0% |
| ≤3 | 19 (3) | 94.7% | 89.2% | 19 (0) | 100.0% |

DFS: disease-free survival; OS: overall survival; irHPC: immune-related histologic phenotype assessment criteria; MPR: major pathologic response; RVT: residual viable tumour; dNLR: derived neutrophil-to-lymphocyte ratio.
