# Supplementary material for: Immune-related histologic phenotype in pretreatment tumour biopsy predicts the efficacy of neoadjuvant anti-PD-1 treatment in squamous lung cancer
Source: BMC Med. 2022 Oct 24;20:403. doi: 10.1186/s12916-022-02609-5 (PMC9594940; doi:10.1186/s12916-022-02609-5)
Supplement: Supplementary file 2 — Additional file 2: Table S2. Different pathologists’ pretreatment prediction of pathologic response after neoadjuvant anti-PD-1 treatment according to irHPC. [file 12916_2022_2609_MOESM2_ESM.docx]

**Table S2 Different pathologists’ pretreatment prediction of pathologic response after neoadjuvant anti-PD-1 treatment according to irHPC**

| **Pathologists** | **Predictive scores** | **cPR/MPR** | **pPR/nPR** |
| --- | --- | --- | --- |
| **C.G.** | ≥2 | 6 | 2 |
|  | <2 | 2 | 5 |
| **L.L.** | ≥2 | 4 | 1 |
|  | <2 | 4 | 6 |
| **P.Y.** | ≥2 | 4 | 1 |
|  | <2 | 4 | 6 |

cPR: complete pathologic response; MPR: major pathologic response; pPR: partial pathologic response; nPR: no pathologic response.
