# Supplementary material for: Immune-related histologic phenotype in pretreatment tumour biopsy predicts the efficacy of neoadjuvant anti-PD-1 treatment in squamous lung cancer
Source: BMC Med. 2022 Oct 24;20:403. doi: 10.1186/s12916-022-02609-5 (PMC9594940; doi:10.1186/s12916-022-02609-5)
Supplement: Supplementary file 3 — Additional file 3: Table S3. Interobserver reproducibility of the irHPC scoring system to predict the pathologic response. [file 12916_2022_2609_MOESM3_ESM.docx]

**Table S3 Interobserver reproducibility of the irHPC scoring system to predict the pathologic response**

| **Measurements** | **Inter-observer reproducibility(N^&^=45)** |
| --- | --- |
| Concordant pairs | 35 (77.8%) |
| Disconcordant pairs | 10 (22.2%) |
| Measure of Agreement(95% CI) |  |
| OPA(%) | 77.8 (76.2-79.8) |
| κ | 0.54 (0.24-0.78) |

^&^N=$C_{3}^{2}$ (the number of comparison pairs of each case)×15 (the number of cases); OPA: overall percent agreement; 95% CI: 95% confidence interval; κ: Fleiss’ kappa.
