# Supplementary material for: Immune-related histologic phenotype in pretreatment tumour biopsy predicts the efficacy of neoadjuvant anti-PD-1 treatment in squamous lung cancer
Source: BMC Med. 2022 Oct 24;20:403. doi: 10.1186/s12916-022-02609-5 (PMC9594940; doi:10.1186/s12916-022-02609-5)
Supplement: Supplementary file 4 — Additional file 4: Table S4. Derived neutrophil-to-lymphocyte ratio (dNLR) supports the negative predictive role of neutrophils. [file 12916_2022_2609_MOESM4_ESM.docx]

**Table S4 Derived neutrophil-to-lymphocyte ratio (dNLR) supports the negative predictive role of neutrophils**

| **Pretreatment complete blood cell counts** | | **cPR/MPR** | **pPR/nPR** |
| --- | --- | --- | --- |
| **dNLR** | ≤3 | 12 | 9 |
|  | >3 | 3 | 7 |

cPR: complete pathologic response; MPR: major pathologic response; pPR: partial pathologic response; nPR: no pathologic response; dNLR: derived neutrophil-to-lymphocyte ratio.
