# Supplementary material for: Immune-related histologic phenotype in pretreatment tumour biopsy predicts the efficacy of neoadjuvant anti-PD-1 treatment in squamous lung cancer
Source: BMC Med. 2022 Oct 24;20:403. doi: 10.1186/s12916-022-02609-5 (PMC9594940; doi:10.1186/s12916-022-02609-5)
Supplement: Supplementary file 5 — Additional file 5: Table S5. The relationship between eosinophils and PD-L1 expression. [file 12916_2022_2609_MOESM5_ESM.docx]

**Table S5 The relationship between eosinophils and PD-L1 expression**

| **Eosinophils** | | **PD-L1 (TPS)** | | ***P*** | **PD-L1 (TPS)** | | ***P*** |
| --- | --- | --- | --- | --- | --- | --- | --- |
| **Parenchyma of tumour** | **Stroma** | **<1%** | **≥1%** |  | **<50%** | **≥50%** |  |
| Presence | Presence | 18 | 27 | 0.343 | 33 | 12 | 0.003 |
| Absence | Absence | 6 | 7 |  | 11 | 2 |  |
| Absence | Presence | 25 | 23 |  | 46 | 2 |  |
| Presence | NA^*^ | 0 | 2 |  | 2 | 0 |  |

PD-L1: programmed cell death-ligand 1; TPS: tumour proportion score.

^*^: no sufficient stroma.
