# Supplementary material for: Immune-related histologic phenotype in pretreatment tumour biopsy predicts the efficacy of neoadjuvant anti-PD-1 treatment in squamous lung cancer
Source: BMC Med. 2022 Oct 24;20:403. doi: 10.1186/s12916-022-02609-5 (PMC9594940; doi:10.1186/s12916-022-02609-5)
Supplement: Supplementary file 6 — Additional file 6: Table S6. Comparison of UICC VIII stages between MPR and non-MPR cases. [file 12916_2022_2609_MOESM6_ESM.docx]

**Table S6 The comparison of UICC Ⅷ stages between MPR and non-MPR cases**

| **UICC Ⅷ stage** | **MPR** | **non-MPR** | P |
| --- | --- | --- | --- |
| Ⅰ | 1 | 3 | 0.178 |
| Ⅱ | 4 | 6 |  |
| Ⅲ | 10 | 7 |  |

UICC: Union for International Cancer Control; MPR: major pathologic response.
