# Supplementary material for: Immune-related histologic phenotype in pretreatment tumour biopsy predicts the efficacy of neoadjuvant anti-PD-1 treatment in squamous lung cancer
Source: BMC Med. 2022 Oct 24;20:403. doi: 10.1186/s12916-022-02609-5 (PMC9594940; doi:10.1186/s12916-022-02609-5)
Supplement: Supplementary file 7 — Additional file 7: Supplemental methods. irHPC: a pretreatment scoring system. [file 12916_2022_2609_MOESM7_ESM.docx]

**Supplementary Methods**

**irHPC: a pretreatment scoring system**

In this scoring system, we should assess the immune status of the parenchyma of tumour [tumour-infiltrating lymphocytes (TILs), eosinophils, neutrophils and macrophages], the parenchyma-stroma interface (irregular interface) and stroma of tumour (dense plasma cells, lymphoid aggregate, proliferative fibrosis, neovascularization, TLSs, mature fibrosis and necrosis):

1. **Tumour-infiltrating lymphocytes (TILs):** Presence of lymphocytes and/or histocytes (dendritic cells and Langerhans cells) in the parenchyma of the tumour. The intensity of the infiltrate was graded as none (0), mild (1+, rare lymphocytes), moderate (2+, focal infiltration of tumour by lymphohistocytic aggregates), or severe (3+, diffuse infiltration).
2. **Eosinophils:** Presence of eosinophils in the parenchyma of the tumour. The intensity of the infiltrate was graded as none (0), mild (1+, rare eosinophils), moderate (2+), or severe (3+, diffuse infiltration).
3. **Neutrophils:** Presence of neutrophils in the parenchyma of the tumour. The intensity of the infiltrate was graded as none (0), mild (1+, rare neutrophils), moderate (2+), or severe (3+, diffuse infiltration).
4. **Macrophages:** Presence of focal infiltration of tumour parenchyma by macrophage aggregates, most often seen as focal granulomas companying scatted lymphocytes plasma cells, located in the margin of the tumor cell nest.
5. **Irregular interface:** Irregular tumour-stroma interface, infiltrated by dense immune cells and/or replaced by regression tissue, mainly focal granuloma.
6. **Dense plasma cells:** Discrete collection of >50 plasma cells in the stroma.
7. **Lymphoid aggregate:** Discrete collection of >100 lymphocytes that does not demonstrate architectural organization or mixture of cell types of a TLS.
8. **Proliferative (new) fibrosis:** Characteristic of tissue repair/wound healing early stage when inflammatory cells release cytokines and growth factors that stimulate proliferation of fibroblast foci. High fibroblast-to-collagen ratio.
9. **Neovascularization:** newly formed small blood vessels, most often seen in a background of tissue-repair.
10. **TLSs:** Tertiary lymphoid structures. Ectopic organized lymphoid node-like structure that includes T cells, activated B cells, high endothelial venules.
11. **Mature fibrosis:** Established scar tissue. Fibroblasts are not as evident and are surrounded by large amounts of collagen. Low fibroblast-to-collagen ratio.
12. **Necrosis**.
